# Supplementary material for: Are carbon emissions trading and green financial instruments synergistic? -Comprehensive quantitative research based on content analysis
Source: PLoS One. 2024 Mar 7;19(3):e0298601. doi: 10.1371/journal.pone.0298601 (PMC10919601; doi:10.1371/journal.pone.0298601)
Supplement: S1 Data — (ZIP) [file pone.0298601.s001.zip › Data/地级市Z_F工作报告.zip!/╣π╬≈/┴°╓▌╩╨/└┤╫╘╣1⁄2╓┌║┼í╛┬φ┐╦╩2╛▌═°í┐_2014.docx]

各位代表：

现在，我代表市人民政府向大会作政府工作报告，请予审查，并请各位政协委员和列席会议的同志提出意见。

一、2013年工作回顾

过去的一年，是柳州发展乘风破浪、昂首奋进的一年。面对复杂多变的国内外形势和繁重艰巨的改革发展任务，在自治区党委、政府和市委的正确领导下，全市上下深入贯彻落实党的十八大和十八届三中全会精神， 化挑战为机遇，化压力为动力，迎难而上，克难攻坚，奋发有为，努力推动经济社会又好又快发展、城市面貌日新月异、人民生活更加改善，柳州发展呈现出勃勃生机，展现出美好前景。

—— — 预计全市地区生产总值突破2000亿元，达2016.9亿元，增长10.0%，工业总产值突破4000亿元，达4020.7亿元，增长12.8%，实现历史性跨越。柳州经济实力进一步壮大，发展基础进一步夯实。

—— — 财政收入285.06亿元，增长9.5%。其中地方财政收入125.12亿元，增长10.2%。财政实力稳步提高。

—— —全社会固定资产投资完成1562.6亿元，增长19.0%。投资拉动有力促进了经济发展。

—— — 城镇居民人均可支配收入24177元，增长9%；农民人均纯收入7557元，增长12%。人民收入水平持续提升。

—— — 城镇登记失业率3.99%，人口自然增长率7.67‰，均控制在自治区下达的指标内；学前教育三年毛入学率77%，比上年提高7个百分点；新农合、城镇职工医保、城镇居民医保参保率均达95%以上；为民办10件实事全面完成。人民生活水平更加改善。

一年来，我们主要抓好以下工作：（一）着力推动工业化、信息化融合发展，加快产业结构调整步伐发展壮大工业经济实力。工业经济克服市场有效需求减弱、企业流动资金紧张、经济下行压力加大等不利影响，实现平稳较快增长，全市规模以上工业产值完成3911.3亿元，增长13%。园区经济快速发展，园区规模以上工业产值突破2000亿元，占全市比重达53%；企业规模进一步壮大，上汽通用五菱、柳钢工业产值迈上600亿元新台阶，上汽通用五菱、东风柳汽汽车产销量分别突破160万辆、20万辆；新增产值过亿元企业34家，总数达到445家。

加快推进产业结构调整。制定实施“创新驱动转型升级 工业再上新台阶”三年计划，加快构建科学合理的现代产业体系。汽车产业优势进一步巩固，产量达186万辆，增长11.4%，产销量在全国排名第五。新产品不断研发上市，五菱宏光S、菱智V3、国内首款“加大号城市SUV”景逸X5等受到市场热捧；柳钢加快发展汽车用板、高强度钢筋等高附加值产品；广西康明斯公司正式投产运营，生产各型号发动机5000台；战略性新兴产业产值突破200亿元，达277亿元，占全市比重7.1%，银海铝业高精度高性能铝及铝合金板带材（一期）等项目竣工投产。

大力提升创新驱动能力。柳州国家级“两化”融合试验区通过验收，柳工、广西糖网跻身“国家级信息化和工业化深度融合示范企业”，“两化”融合水平进一步提升。柳南装备制造（工程机械）产业示范基地列为“国家新型工业化产业示范基地”，柳州成为全区唯一拥有两个国家级产业示范基地的城市。高新技术企业加快发展，新增国家高新技术企业16家，已达到129家，新增广西创新型企业5家、自治区工程技术中心4家，已分别达到16家、25家，欧维姆迈入“国家技术创新示范企业”行列。企业具有自主知识产权的产品产值超过工业总产值的1/3。柳钢、柳工、上汽通用五菱、东风柳汽、鱼峰水泥、柳化荣获自治区“先进质量管理方法标杆企业”。

深入推进汽车城建设。上汽通用五菱宝骏基地发动机四期、N111（新五菱之光）项目竣工投产，生产汽车18万辆。东风柳汽乘用车新基地（一期）四大工艺车间抓紧施工，涂装车间和总装车间建设完成。引入24家零部件企业进驻花岭产业园，与联合电子、福耀玻璃签订了投资协议，经深入洽谈，耐世特、法士特等知名企业有意落户汽车城。成功举办第二届中欧（柳州）汽车产业高峰论坛，启动中欧合作产业园建设。国家汽车质量监督检验中心（柳州） 、上海交通大学汽车先进制造技术联合实验室等项目顺利推进。

扎实开展节能减排。全市规模以上工业企业万元工业增加值能耗持续下降，“十一五”期以来年均下降8.96%。主要污染物减排工作扎实推进，顺利完成自治区下达的年度减排项目。柳电公司进行两台机组脱硝改造，2号机组脱硝工程投入运行，柳钢焦化煤调湿工程 、鱼峰水泥4#生产线脱硝工程建成使用。积极发展循环经济，鹿寨经济开发区列入国家循环化改造示范试点园区，电镀工业园开工建设。继续淘汰落后产能，淘汰水泥落后产能48.8万吨 、造纸落后产能2.4万吨。

（二）着力加快推进城镇化、农业现代化，统筹城乡新发展积极推进农业现代化。全年实现农林牧渔业总产值259亿元，增长5.2%。

深入实施“农民人均纯收入倍增计划”，加快“倍增计划十大产业工程”建设，完善和新建5万亩优质稻 、双季葡萄 、标准茶园 、蔗种良繁 、高效桑蚕等优质高产示范基地，新建274个生猪 、禽蛋 、特色水产规模化养殖场。粮食生产再获丰收，水果产业优化升级，蔬菜产业加快发展。新一轮“菜篮子”工程深入推进，“菜篮子”产品自主保障与供应能力不断增强。现代农业产业集群发展取得新突破，葡萄种植面积较大幅度增加，产量增长20%，蛋鸡 、旱鸭 、双季莲藕和香葱生产基地规模不断壮大，培育了一批现代化 、智能化食用菌龙头企业及食用菌专业村屯。积极推动农业产销对接及开发合作，成功举办“2013柳州名特优农产品（上海）推广周”。农业科技创新能力不断提升，“国家茶产业工程技术研究中心柳州分中心”、“水生蔬菜西南繁育中心”建设顺利推进，柳城县列入国家级“出口水果质量安全示范区”。积极推进甘蔗生产全程机械化及高效节水灌溉种植，全市综合农机化水平达45%，在全区排名第一。

统筹实施城乡建设。加快建设“一小时经济圈”和“两小时经济带”交通路网，三江至柳州高速公路建设顺利推进，柳州至江口至导江二级公路 、国道209融安绕城公路 、国道209改道工程三江至林溪公路等全面启动。继续推进柳江鹿寨与市区同城化发展，编制完成《柳江鹿寨与市区同城化建设衔接规划》，启动实施市 、县同城化建设。城镇建设水平不断提升，积极推进柳北区沙塘镇“全国发展改革试点城镇”建设，柳东新区雒容镇 、三江县丹洲村和融水县雨卜村申报自治区级特色名镇名村。农业转移人口市民化有序推进，全市城镇化率提高至59.45%。

持续改善农村生产生活条件。落久水利枢纽工程项目建议书获国家发改委批复，洋溪水利枢纽工程前期工作顺利推进，16个水库移民新村建设项目全部完工，313座病险水库除险加固工作扎实开展。中小河流治理 、农田水利建设加快推进，新增恢复改善灌溉面积11.2万亩，新建渠道410公里。开工建设373处农村饮水安全工程项目，有效解决了18.4万农村人口饮水安全问题。

农村路网建设深入推进，行政村通水泥路率达到70%。2011-2012年农村电网改造升级工程通过整体验收。有效推进矿山废弃地开发整理，开展“小块并大块”耕地整治 、“整县推进”高标准基本农田土地整治以及农村土地综合整治，柳江思荣锰矿区 、柳南帽合村 、柳北大仙村建设用地增减挂钩深入推进。

“美丽柳州·清洁乡村”活动深入开展，清洁家园 、清洁水源 、清洁田园成效明显，农村人居环境持续改善，生态建设与乡风文明水平进一步提升。

加快发展县域经济。坚持分类指导，分层推进，因地制宜推进县域产业园区建设，实行差别竞争 、错位发展，不断提升县域经济水平。六县实现地区生产总值546.7亿元，增长9.8%；规模以上工业总产值492.2亿元，增长15.4%；财政收入40.45亿元，增长10.6%；全社会固定资产投资572.5亿元，增长27.2%。

（三）着力加快现代服务业发展，积极打造区域性服务中心大力推进重点项目建设。积极引进万达 、华润 、恒大 、华侨城 、海雅 、红星美凯龙 、毅德等知名企业落户柳州，进一步提升我市服务业发展水平，优化三次产业结构。推动步步高购物广场开业运营 、乐和城和金绿洲主题商业楼主体竣工。以列入全国二级物流园区布局城市为契机，加快建设区域性商贸物流 中心，鹧鸪江钢铁深加工及物流产业园（一期） 开业运营，广西长乐物流园（一期） 、桂中海迅柳北物流基地（一期）等项目竣工。广西医药物流中心 、新柳邕农贸市场等现代大型物流项目加速推进。全年服务业实现增加值579.1亿元，增长8.5%。

稳步提升城乡居民消费。组织开展“消费购物节”等各类促进消费活动56个，消费额达30亿元。全年举办各类会展35个，拉动消费20多亿元，会展业拉动经济效果初步显现；柳州国际会展中心主体功能区建成使用，具备承接举办各类大型展会能力。商品流通网络和农产品批发市场体系不断完善，新建和改造社区微型超市50家 、城乡集贸市场22个 、万村千乡市场工程配送中心3个 、农家店46个。全社会消费品零售总额758.5亿元，增长14.6%。全年居民消费价格总水平上涨1.9%，控制在年初确定的4%目标之内。

扎实推进旅游名城建设。抓住自治区召开旅游大会的有利时机，精心策划一批重大旅游项目，列入自治区统筹推进的重大项目12项，列入自治区旅游重点项目55项，列入自治区领导重点联系的旅游项目2项，融水县 、三江县列入自治区创建特色旅游名县。旅游基础设施不断完善，百里柳江等一批重点旅游景区建设扎实推进，园博园 、石门仙湖景区 、柳城知青城被评为国家4A景区，丹洲景区 、山岔湾度假山庄被评为五星级乡村旅游区，月岛湖度假区被评为广西五星级农家乐，新增五星级酒店1家，四星级酒店2家。柳州荣获“2013中国最佳休闲城市”。全年实现旅游收入186亿元，增长21%。

加快发展金融业。全市年末金融机构本外币各项存 、贷款余额分别达2327.78亿元和1640.57亿元，增长21%和17%，中小企业贷款增量连续三年排名全区第一。全年新增各类金融机构3家，总数达75家。金融创新能力稳步提升，广西第一支18.6亿元保险债权资金落户柳州，成功发行第一支区域集优票据。金融生态环境不断优化，直接融资渠道不断拓宽，柳州成为国开行重点支持城镇化建设试点城市和广西中小微企业创新融资模式试点城市，全市小额贷款公司由上年的19家增至42家，累计发放贷款突破100亿元，柳州银行监管评级达到全国城商行序列最好等级，成为广西唯一获此殊荣的银行。

保持房地产市场健康稳定。认真贯彻执行国家和自治区调控政策，推动房地产市场持续健康发展。在市区范围内开展商品房预售方案审核工作，强化房地产市场监管，积极推进一系列规范性文件出台，推进行业管理制度化。商品住房供给和消费平稳，销售商品房面积307.5万平方米，房地产土地市场总体平稳，商品房价格涨幅控制在目标范围之内。

（四）着力建设美丽柳州，打造生态宜居城市全面加快柳东新区建设。新区基础设施不断完善，环岭东路实现通车，打通整车基地与零部件产业园的交通主动脉，双仁路 、智和路 、新柳大道延长线等项目加快推进；供水管道工程实现通水，官塘污水处理厂投入试运行。城市配套服务功能日益完备，柳东实验中学 、实验小学建设及柳职院柳东校区（一期） 、广西科技大学汽车学院前期工作有序推进，市儿童医院 、华侨城文化旅游科技体验园开工建设。

努力打造对外交通新优势。衡柳 、柳南高铁开通运营，柳州步入高铁时代，柳肇铁路列入“十二五”期国家西部大开发战略重点项目，柳韶铁路前期工作有序开展。柳州至梧州 、桂林至三江 、三江至柳州等5条高速公路建设稳步推进。阳和作业区（一期）工程和鹧鸪江作业区1#、4#泊位投入运营，柳江二级航道整治工程启动。柳州白莲机场停机坪及配套设施扩建工程动工建设。

持续提升城市建设和管理水平。地王国际财富中心主楼封顶，风情港主体完工，金沙角顺利建成，城市新地标风采初显。文惠桥改造完成，莲花立交桥 、广雅大桥工程竣工通车，北外环 、西外环和南外环城市快环路系统基本建成通车，城市道路交通网络进一步优化。城市快速公交系统（BRT）一号线通车运营，公用自行车租赁系统（一期）设45个点 、投放自行车1300辆，河东综合交通枢纽项目加快推进，火车站站前广场改造开工建设，柳州成为国家“公交都市”示范工程第二批创建城市。申报国家“智慧城市”试点取得成功。鹧鸪江大桥获得“鲁班奖”。开展严厉打击违法占地违法建筑“风暴行动”，累计拆除“两违”面积233.8万平方米，得到国家住建部 、自治区领导充分肯定。

加快生态宜居城市建设步伐。深入实施“花园城市”、“绿满龙城”等绿化美化工程，全市新增绿地300公顷，种植各类苗木 、植物345万株，森林覆盖率达到64.36%，市区绿化覆盖率达到45.7%，柳州荣获“全国绿化模范城市”。着力推进马鹿山公园奇石文化项 目群 、鹅山公园和雀山公园景区升级改造等工程建设，群众休闲健身场所日益完善。率先在全区建成日供能力30万吨的地下水备用水源，开工建设古偿河第二水源，有效保障了公共供水安全。

加快推进城郊污水收集系统 、沙塘片区污水处理等工程建设，污泥处理形成每日300吨的能力。全面开展环境安全隐患大清查大整治行动，共清查企业1582家，停产整治77家，拟关闭取缔59家。全年柳江河饮用水保护河段继续保持国家地表水Ⅲ类以上水质标准，市区环境空气质量达到国家二级标准。

（五）着力改善民生，让人民群众共享更多发展成果扎实推进民生改善工程。深入实施保障性安居工程，保障性住房完成投资35.6亿元，新开工建设13353套，基本建成15147套，分配入住住房6258套；职工危旧房集中区改造完成投资17.44亿元，施工面积137.26万平方米（含新建及续建），新建住房2296套；积极推动柳北白沙村 、柳南帽合村 、鱼峰水南村 、柳东新区下窑村等城中村改造，改善群众居住生活状况；农村危房改造工程5120户续建项目全部完工，新增1.3万户改造工程全部开工；三江县第二期少数民族村寨防火改造工程稳步推进。九年义务教育农村学生免费午餐惠及1061所学校，受益学生17.98万人。

老有所养工程稳步开展，10万平方米集中养老区开工建设。农村扶贫开发攻坚战扎实推进，连续两年在自治区考核中评为A等级。

不断健全社会保障体系。初步建成覆盖城乡居民的社保体系，连续9年上调基本养老保险待遇，企业职工基本养老保险待遇人均月增资194.13元，月人均养老金达1721元，比2012年增长12.4%；市区城市低保标准提高至每人每月390元，农村低保标准提高至每人每年2400元。救助城乡低保对象186.6万人次，救助受灾人口10.4万人。实施北部三县农房保险工程，惠及人口60多万户。积极推进城乡居民大病保险试点工作。

实施就业援助工程，以创业促进就业。以高校毕业生 、农村劳动力 、下岗失业人员和就业困难人员为重点群体，开展就业援助工作，全市城镇新增就业人数5.91万人，新增农村劳动力转移就业5.65万人，高校毕业生就业率达85%以上。出台新的创业小额担保贷款办法，进一步完善创业带动就业政策，新发展微型企业1812户，总数达5153户，建成大学生创业园一期创业孵化基地，首批40个创业项目进展顺利。

（六）着力深化改革与扩大开放，不断增强发展动力与活力稳步推进各项改革。深化国资国企改革，国有资产实现保值增值，市属国有及国有控股企业资产总量突破2500亿元，达2568亿元，增长24.5%。政府投融资平台公司市场化运作能力不断增强，全年融资348.37亿元，增长56%，创历史新高。积极促进非公有制经济发展，全市私营企业达2.9万户，从业人员33.1万人，分别增长21.12%和16.08%。医疗卫生体制改革取得新进展，文化及农 、林等领域改革不断推进。

进一步拓展对外开放合作。主动参与北部湾经济区开放开发，成功举办第三届中国—东盟（柳州）汽车 、工程机械及零部件博览会，吸引150家国内外企业参展，成交2.33亿元，通过市场化运作提升了展会专业化 、品牌化和国际化水平。促进西江经济带开发合作，推动柳来河一体化发展。企业“走出去”力度加大，境外投资3511.1万美元，增长31.89%。全年完成进出口总额28.6亿美元，其中出口9.1亿美元。

招商引资成效明显，围绕重点产业布局，积极引进一批战略性新兴产业 、现代服务业 、现代农业和文化旅游业等领域项目，招大引强实现新突破。引进市外境内到位资金（含续建项目）600亿元，利用外资（全口径）实际到位资金3.6亿美元。

（七）着力发展社会事业，保持社会大局和谐稳定深入实施文化惠民工程。大力宣传党的十八大及十八届三中全会精神，扎实推进社会主义精神文明建设。稳步推进文化重点项目建设，中国柳江人遗址博物馆正式动工，刘三姐文化娱乐中心开业，柳州艺术馆建成使用，成为我市首个书画艺术专业展览场馆，中国国家画院柳州创作基地挂牌成立。柳州工业博物馆获第十届全国博物馆十大陈列展览精品优秀奖，24家博物馆被列入广西首批百家博物馆单位。文艺创作取得新成果，原创舞蹈《仫佬仫佬背背抱抱》荣获第九届中国舞蹈“荷花奖”金奖，三江县侗族大歌《侗不离酸》 、柳城县“百村百戏”荣获第十届中国艺术节“群星奖”，《八桂大歌》升级版成功首演。成功举办2013年中国柳州国际水上狂欢节 、F1摩托艇世界锦标赛中国柳州大奖赛 、水上摩托世界锦标赛等重大赛事，柳州知名度和影响力不断提升。

推进科技创新与招才引智。成功承 办第二届广西发明创造成果展览交易会，积极推进科技成果转化。全力推进发明专利倍增计划，共申请发明专利1187件，获授权专利513件。在全区率先开展专利质押融资工作，实现了专利资本化，龙杰汽配等7家企业通过专利质押融资获得银行贷款8300万元。

柳州荣获“全国科技进步考核先进市”。

招才引智渠道不断拓宽，柳化获准设立博士后科研工作站，全市博士后科研工作站增至8家。深入实施第三届“优秀人才接触柳州”计划和“双百人才工程”，全市18家人才小高地建设扎实推进，目前拥有各类高层次人才2052人，其中院士9人。

加快发展教育事业。启动实施市区义务教育均衡发展学校基本建设三年攻坚计划，三年内投入19亿元实施86项义务教育学校新建和改扩建工程。加快发展学前教育，完成学前三年行动计划新建和改扩建公办幼儿园项目60所。

普通高中教育质量稳居全区前茅，全市自治区示范性普通高中增至14所。职业教育影响力不断扩大，校企间“订单输出”、集中办学 、资助贫困学生等形成了独特的柳州经验。高等教育取得新进展，广西工学院更名为广西科技大学。

持续提高医疗卫生保障能力。进一步扩大基本药物制度实施范围，在113个公办基层医疗卫生机构 、649个村卫生室配备和使用基本药物，实行零差率销售。在全区率先全面启动县级公立医院综合改革，率先推行“乡村移动医疗”服务模式，不断完善农村三级服务网络。城市社区卫生服务实现全覆盖，全民健康“一卡通”项目正式启动，艾滋病等重大传染病得到有效防控。深入开展创建“国家卫生城市”活动，相关工作通过国家专家组暗访和技术评估。

柳铁中心医院1号住院大楼竣工，柳江 、融安 、融水三县人民医院扩建完工投入使用。

加强和创新社会管理。市长“企业接待日”、“市民接待日”进一步制度化长效化，共接访企业 、群众各种利益诉求100多件，努力为基层 、为企业 、为群众排忧解难。“平安柳州”建设扎实推进，“天网”工程新建成2334个高清监控点，市区监控点达到5100个；建立“一村一警”警务机制，六县农村地区110接报警案件同比下降39.3%。

继续完善社区网格化管理服务，加快社区信息化建设。“大人口”信息化建设应用实现共建共享，制定出台计划生育特殊家庭扶助办法，免费孕前优生健康检查实现城乡全覆盖。扎实做好食品药品监管，群众饮食用药安全保障水平进一步提升。安全生产监督管理进一步强化。民族团结进步事业取得新成就，社会大局保持和谐稳定。

（八）着力加强政府自身建设，进一步优化发展环境进一步加强作风建设。认真践行党的群众路线，坚决反对“四风”，严格执行“八项规定”，扎实开展公车专项治理，柳南区率先进行党政机关公务用车制度改革。政务服务体系不断完善，市 、县（区） 、乡镇（街道） 、村（社区）四级政务服务体系建设加快推进，全年各级政务服务中心办结事项133.81万件；推进行政审批制度改革，取消 、调整合并 、转变管理方式行政审批项目100项，占所有审批事项的17.99%；积极推动重大项目审批绿色通道建设，绿色通道办件1528件；不断完善投资企业帮扶机制，协助企业完善项目手续并联审批办件3345件。扎实开展第七届“中小企业服务月”活动，为500多户企业提供咨询 、科技 、融资 、审批等多种形式的服务，取得良好效果。机关绩效考评和绩效管理深入推进，柳州被评为“2012年度广西壮族自治区绩效考评优秀市”。

进一步加强民主法治建设。坚持政府重大事项向人大及其常委会报告 、向政协通报制度，自觉接受市人大的依法监督和市政协的民主监督。人大代表议案 、建议和政协委员提案答复率达100%。深入推进“阳光信访举报平台”、“非公有制经济发展投诉受理平台”、“三级网络舆情监控平台”建设，拓宽了社会公众监督政府渠道。“六五”普法和“法治柳州”建设深入开展，法制宣传力度不断加大，人民调解工作深入推进，各级人民调解组织调解矛盾纠纷1.9万件。

进一步加强党风廉政建设。认真落实党风廉政建设责任制，以制度化建设为核心，不断完善廉政风险防控机制。

落实集体廉政谈话机制，强化提醒教育。开展述责评议，强化“一把手”阳光用权。加快公共资源交易体制改革，成立市公共资源交易中心，建立完善公共资源交易市场管理体制和监督机制。

围绕重点领域开展专项执法监察8项，取消考核 、评比及节庆 、论坛 、展会等项目49项；扎实推进基层廉政建设，对97个乡镇 、990个村及涉农社区进行了清产核资。

过去一年，我市人事 、编制 、外事侨务 、工商 、税务 、技术监督 、统计 、国家安全 、决策咨询 、档案 、人防 、气 象 、粮食 、宗教 、老龄 、修志 、防汛抗旱 、防震减灾 、哲学社会科学等各项工作都取得了新的成绩。

各位代表，过去的一年，我们取得的成绩来之不易，这是市委正确领导及市人大 、市政协监督支持的结果，是全市人民精诚团结 、共克时艰的结果，是社会各界鼎力支持 、积极奉献的结果。

在此，我代表市人民政府，向全市人民，向驻柳人民解放军 、武警部队官兵，向各民主党派 、工商联 、人民团体及海内外所有关心和支持我市发展的各界人士，致以崇高的敬意和衷心的感谢！在总结成绩的同时，我们也清醒地认识到存在的困难与问题，主要是：支撑工业经济持续较快增长的动力依然不足；农业产业化发展步伐有待加快；服务业总体发展仍然滞后，服务业发展水平及发展层次有待提高；城乡 、县域之间发展不平衡，部分群众生活还比较困难；社会建设与人民群众的期待仍有差距，社会管理水平有待进一步提高，等等。我们要引起高度重视，采取有效措施，认真加以解决，把全市改革发展各项事业继续推向前进。

二、2014年工作总体要求和目标任务

今年，是全面贯彻落实党的十八届三中全会精神的开局之年，是珠江—西江经济带发展规划启动实施之年。

综观宏观形势，我们仍处于可以大有作为的重要战略机遇期，具备许多有利条件和积极因素，也面临较多困难和挑战。当前，国内外经济发展不确定因素较多，受国际金融危机深层次影响，世界经济体低速增长的态势仍在延续，国内经济步入增速放缓期，转方式 、调结构 、稳增长 、促发展任务繁重艰巨。然而，历史也为我们提供了难得的发展机遇，党的十八届三中全会作出了全面深化改革的决定，珠江—西江经济带发展规划即将上升为国家战略，将进一步激发我市发展的内生动力与活力。我们要坚定信心，沉着应对 、抢抓机遇 、加快发展，不断谱写柳州改革发展的新篇章！今年政府工作的总体思路是：全面贯彻落实党的十八大和十八届三中全会精神，贯彻落实中央和自治区经济工作会议及市委十一届十次全会精神，按照“经济升级 、城市转型，全面发展 、富民强柳”的总体发展思路，大力实施“实业兴市，开放强柳”战略，按照稳中求进的工作总基调，以扎实开展科学发展提升年 、改革创新突破年 、作风建设提升年为抓手，稳增长 、调结构 、促改革 、惠民生 、优环境，加快打造柳州经济升级版，为在广西率先全面建成小康社会 、建设“五美五好”柳州打下坚实基础。

今年我市经济社会发展的主要预期目标是：地区生产总值增长10%；工业总产值增长14%；全社会固定资产投资增长15%；财政收入增长10%；全社会消费品零售总额增长15%；城镇居民人均可支配收入增长10%；农民人均纯收入增长13%；单位生产总值能耗 、主要污染物排放控制在自治区下达指标以内；城镇登记失业率控制在4.5%以内；人口自然增长率控制在8.5‰以内。

做好今年的工作，要着重把握好以下几个方面：坚持准确把握新形势新变化。党的十八届三中全会对全面深化改革作出了系统部署，涉及经济 、政治 、文化 、社会 、生态文明及党的建设各领域，将释放出巨大改革红利，促发展 、促转型 、促民生改善。自治区党委十届四次全会提出了《关于贯彻落实〈中共中央关于全面深化改革若干重大问题的决定〉的意见》，对广西全面深化改革作出总体部署，明确了主攻方向和重点要求，坚定不移地全面推进改革，用改革的精神 、思路 、办法统筹推进各项工作，必将在经济社会发展上赢得主动。自治区党委结合广西实际，提出了加快“两个建成”的奋斗目标，在全国区域发展大格局中提升了广西的定位和作用，进一步激发了全区上下竞相发展的热情和干劲。同时，珠江—西江经济带发展规划即将上升为国家战略 、广西启动实施“双核”驱动 、市委十一届十次全会明确实施“实业兴市，开放强柳”战略，这将为柳州发展带来新机遇新动力。准确把握我们面临的新形势新变化，有利于稳中求进，促进柳州经济社会又好又快发展。

坚持把改革贯穿于经济社会发展各领域各环节，努力取得新突破新成效。

今年是全面深化改革开局启动之年，群众对改革关注度高 、期望值大，要顺应人民群众的新期盼，坚持顶层设计和“摸着石头过河”相结合，整体推进和重点突破相促进，在中央确定的框架内，合理布局柳州全面深化改革的战略重点 、优先顺序 、主攻方向 、工作机制 、推进方式和时间表 、路线图，科学谋划全面深化改革各项工作。对方向 明 、见效快 、有权自主操作的改革，要尽快启动 、全力推进；对涉及面广 、需要中央和自治区决策的改革，要及时跟进中央和自治区的新部署新要求，谋定而后动；对还未达成共识 、条件尚不具备但又必须推进的改革，要大胆探索 、先行先试。要牢牢把握全面深化改革的主导权主动权，准确有序协调推进各项改革，不断取得新成效。

坚持围绕打造柳州工业升级版，加快结构优化、转型升级。要立足于工业是柳州命脉这一最大实际，充分把握“强产业 、夯基础 、优结构”是工业转型升级的重要支撑，抓紧实施“创新驱动 转型升级工业再上新台阶”三年计划，做好调整产业结构这篇大文章，充分发挥汽车 、冶金 、机械三大支柱产业牵引带动作用，改造提升化工 、建材 、制糖 、日化等传统优势产业，努力构建科学合理的现代产业体系。要千方百计扩投资上项目，引进工业大项目 、好项目，以项目建设助推产业结构调整，推动工业产业 、企业 、产品从传统向现代 、从低端向高端 、从低附加值向高附加值 、从低效益向高效益升级。要抢占发展先机，全面加快新材料 、新能源汽车和节能环保 、先进装备制造和电子信息 、生物和制药等新兴产业发展，加快发展以三大支柱产业新兴化为重点的智能制造 、新一代信息技术等产业，在技术研发 、市场培育 、信息化建设等方面进行重点扶持，努力将战略性新兴产业打造成为经济增长新引擎，为柳州工业发展提供新的支撑和动力。

坚持把发展第三产业放在更加突出的位置，大力发展现代服务业。把发展现代服务业作为调整产业结构的主攻方向，结合城市改造和发展方向，围绕建设区域性金融中心 、信息中心 、服务中心，将发展现代服务业与提升传统优势服务业有效结合，进一步优化服务业空间布局，加快建设大型现代服务业综合体，努力构建现代服务业集聚区。融合柳州“桂中商埠”的历史文脉与现代都市文明，大力发展生产性服务业 、消费性服务业和新兴服务业，把现代物流 、商贸及零售 、金融服务 、中介咨询 、电子商务等业态做大做强。要结合全区旅游发展大会的工作部署，突出抓好旅游基础设施 、旅游景点和服务设施建设，打造“百里柳江”观光运动休闲旅游 、“百里风情”民俗文化旅游 、现代工业文明体验旅游，充分彰显融自然山水 、民族风情和工业文化于一体的柳州旅游特色。

坚持进一步扩大开放，合作共赢促发展。要牢牢把握当前开放主导的新特征，把开放作为经济转型升级的重要拉动力，以开放促转型 、以开放促创新 、以开放促发展。要进一步拓展对外开放的广度与深度，围绕重点产业布局和做大做强产业集群，创新招商模式，努力在引进项目 、资金 、技术的质量 、层次和规模上取得新突破。要加强区域交流与合作，推动珠江—西江经济带 、柳来河一体化等区域协作，在交通网络 、产业分工协作和市场体系建设等方面实现共建共享。要围绕打造柳州对外开放新高地，充分把握中央全面深化改革和自治区实施“双核”驱动战略的政策利好，最大限度地发挥政策效应，增强柳州投资吸引力 、集聚影响力，以大开放促进大发展。

坚持立足当前、着眼长远、把握全局、统筹兼顾。切实解决好当前经济社会发展面临的突出问题，抓住关键环节实现突破；注意把握全局统筹规划，从长远和战略高度谋划发展。大力发展现代农业。紧密结合当前全面深化改革的要求，深入研究探索柳州农业发展的新体制 、新机制，努力在新型农业经营体系建设 、农业资源要素配置 、农村产权制度改革等方面取得新进展，重点培育壮大农业产业化龙头企业，以龙头企业的带动进一步提升农业产业化发展水平。大力推进新型城镇化建设。坚持“以人为本 、集约高效 、绿色发展 、四化同步 、城乡一体 、多元特色”的原则，完善主城区功能 、拓展发展空间，实施大县城战略，发展特色小城镇，构建以柳州为核心的桂中城镇群。大力发展非公有制经济。加快实施非公有制强优企业培育计划，集中政策 、资金 、人才等优势资源，培育扶持一批带动力强 、辐射面广的非公企业，推动非公有制经济实现跨越发展。大力发展教育事业。深化教育领域综合改革，促进各类教育协调发展，深入实施义务教育均衡发展学校基本建设三年攻坚计划。全面贯彻即将召开的自治区教育大会精神，不断提高教育发展质量和水平。大力推进区域性综合交通枢纽建设。以更加前瞻性的思维和扎实有效的举措，推进柳州机场国际化发展 、柳肇铁路早日开工建设 、柳江航运能力提升，加快打造区域性综合交通枢纽。紧密结合“十二五”发展规划，着眼柳州长远发展，从全局和战略的高度科学谋划各项事业，全面加强经济 、政治 、文化 、社会和生态文明建设，把柳州发展的宏伟蓝图一步步变成美好现实。

为实现今年工作目标任务，应主要抓好以下工作：

（一）积极开展改革创新突破年活动，奋力开创柳州改革开放新局面深入贯彻落实党的十八届三中全会精神，深入贯彻落实自治区党委、市委关于改革的决策部署，把改革贯穿于经济社会发展各个领域各个环节，使市场在资源配置中起决定性作用和更好发挥政府作用，促进柳州科学发展。

积极推进经济体制改革。深化国资国企改革，创新国有资产监管方式，提升国有企业市场竞争力。推动政府性投资公司市场化改革和转型升级，不断提高发展的质量和效益，实现国有资产保值增值。大力发展以现代产权制度为基础的混合所有制经济，积极探索非公有制经济规模化、特色化、专业化、高新化转型升级之路，从财政扶持、税收优惠、用地保障等方面扶持非公企业发展，大力推进全民创业，充分激发民资民智民力的活力和创造力。

着力推进要素市场改革。深入改善金融生态环境，提升地方金融服务和监管能力，加快培育西江经济带金融中心。充分发挥政府性投资公司投融资功能，优化融资结构，控制融资成本，提升投资综合效益。加大企业信用管理信息系统和个人征信信息系统的应用，扎实推进地方信用体系建设。加快投融资体制改革，发挥好金融对产业升级 、城市转型的支撑作用，提高直接融资比例。促进金融机构开展中小微企业和民生金融，扩展金融服务的普惠面。以农业产业化、新农村建设和农村产权制度改革为切入点，鼓励农村金融创新，推进农民信用建设。优化城乡土地资源市场化配置，稳步实施农村土地流转、集体林权制度配套改革，提高农村集体和农民在土地增值收益中的分配比例，探索建立城乡统一的建设用地市场。积极推进人才体制机制改革，构建高层次人才优势，加快建立各类人才的培育、引进、使用和激励机制。

全面推进社会各项改革。加大社会事业改革创新力度，进一步优化社会事业领域资源配置。深化教育体制改革，完善城乡义务教育资源均衡配置体制机制，加快现代职业教育体系建设。深化社会保障体制改革，完善城乡均等的公共就业服务体系，提升保障水平。深化文化体制改革，推进国有经营性文化单位转企改制，鼓励非公有制文化企业发展。深化医药卫生体制改革，推进公立医院改革和基层医疗卫生机构综合改革，健全城乡基层医疗卫生服务运行机制。

大力发展开放型经济。加快融入珠江—西江经济带开放开发，深化与粤港澳台在战略性新兴产业、现代服务业、文化旅游、特色农业和城市大型综合体等领域的合作，积极承接东部产业转移，主动对接先进生产力。加快企业“走出去”步伐，扩大汽车及零部件、工程机械、钢铁等优势产品出口，支持企业建立境外市场营销网络和售后服务体系，提升企业参与国际竞争的实力。

积极营造开明、开放的投资环境，提高招商引资的质量和水平，大力引进“央企入柳”、“民企入柳”。

（二）坚持创新驱动、转型升级，努力打造柳州工业经济升级版围绕开展科学发展提升年活动，以提高经济发展质量和效益为中心，推动工业经济转型升级。

深入实施“创新驱动转型升级工业再上新台阶”三年计划。以推动产业转型升级为主攻方向，年度投资162亿元实施百项工业结构调整重点项目建设，开工建设柳工全球研发中心、柳州菱特V6汽车发动机、五菱集团年产70万套乘用车零部件等项目，加快推进柳工压缩机生产基地、柳州卷烟厂百万箱技术改造、新宇公司50万锭纺纱能力提升改造等项目建设，竣工投产中成化工年产10万吨保险粉等项目。促进强优企业、中小企业加快发展，力争上汽通用五菱、柳钢主营业务收入突破700亿元，柳工、东风柳汽、五菱有限公司突破200亿元，新增过亿元企业、规模以上企业各30家。加速培育战略性新兴产业，设立战略性新兴产业发展资金，实施战略性新兴产业企业成长计划，重点扶持一批关联性强、成长性高、发展前景好的高新技术企业，支持五菱汽车公司中小型纯电动商用车产业化、腾龙煤电年产3000套系统软件、圣特药业壮药研发生产等项目建设，实现战略性新兴产业产值占全市比重达到8.5%。加快园区发展，突出园区特色，完善园区功能，提升发展水平，做大做强园区产业，实现全市工业园区规模以上工业总产值增长15%以上。加快转变工业发展方式，大力发展低碳经济，促进节能降耗、淘汰落后产能、鼓励清洁生产，投入20.5亿元实施10个生态环保和节能减排项目，重点抓好柳钢球 团烟气脱硫技术改造、鹿寨热电联产、电镀工业园等项目建设。

推动汽车城建设提速增效。加快建设汽车城整车基地，发挥集聚效应，充分释放产能，推动汽车城发展再上新台阶。启动上汽通用五菱宝骏基地试车跑道和研发中心建设，升级改造CN200、GP30汽车，新开发GP50、GP60乘用车及涡轮增压发动机项目；推进东风柳汽乘用车基地（一期）冲压、焊接车间完成建设，实现四大工艺车间全线竣工投产，新开工乘用车基地（二期）、商用车基地、发动机、研发中心等项目；加快五菱集团零部件及新能源汽车基地建设。同步推进联合电子、福耀玻璃、玲珑轮胎等汽车零部件项目建设。加快建设重庆工业园、国家汽车质量监督检验中心（柳州）和国家进出口汽车检测重点实验室（柳州），启动北斗信息产业园建设。

全面建设创新型城市。坚持以创新驱动引领产业升级，全市设立技术研发机构的企业研发投入占销售收入（R&D）比重努力达到2.2%。深化国家级工业化和信息化融合试验区建设，建成30个以上“两化”融合示范项目、示范企业、公共服务平台，建设阳和工业新区“广西两化融合示范园区”。继续实施广西第五轮创新计划，投资30亿元实施160项工业创新项目，年内培育自治区级企业技术中心（研发中心）8家。引导企业加大技术创新投入，重点建设柳工工程机械核心铸件研发等创新项目。进一步完善技术创新体系，推动河西工业园区升级为自治区级高新区，开展产业技术创新战略联盟，培育工程机械产业示范园区公共服务平台。

积极引导企业生产流程再造，进一步优化生产流程，增强企业市场竞争能力。

实施质量强市战略，开展质量基础、质量提升和质量安全工程建设，努力创建“全国质量强市示范城市”。建立以企业为主体的产学研联合体，加快柳东新区科技园、市科技创新服务中心建设，促进中南大学国家重金属污染防治重点实验室、武汉理工大学国家级机电工程实验实训中心落户柳州，打造区域产学研合作创新示范基地。

（三）推进新型城镇化、农业现代化协调发展，构建城乡一体、互惠互动新格局贯彻落实中央农村工作会议精神，以国家推进新型城镇化为契机，通过城镇带动农村，工业反哺农业，全面推进城乡一体化发展。

大力发展现代农业。严格保护耕地，稳定粮食播种面积，提高耕作技术，推广高产优质水稻，保持粮食稳定生产。推进甘蔗生产良种化、机械化、水利化、契约化，发展壮大双季葡萄、食用菌、林下种养、稻田养鱼等特色产业。年内创建高效特色种植业基地10万亩、“万元养殖小区（村、场）”200个、林业经济基地10万亩，打造“万元村屯”30个、“十万元示范农户”200户。深入实施“菜篮子”工程，新建和完善蔬菜保障性基地1万亩，新建规模化畜禽养殖场170个。推进农产品质量安全工程，抓好农产品标准化生产和“三品一标”认证。开展农产品加工、冷链、仓储、物流一体化建设，培育名特优农产品品牌。实施农业机械化创新示范工程，力争全市农机化综合水平达49%。

进一步增强农村经济活力。深入推进“农民人均纯收入倍增计划”，健全财政支农投入增长机制，优化财政支农投入结构，落实各项强农惠农富农政策，提高农业综合生产能力。积极发展农民股份合作，鼓励农民专业合作组织发展。有序推进农村土地制度改革，探索农村集体经营性建设用地出让、租赁、入股的体制机制，稳步推动农村产权流转交易健康有序进行，着力推进农村土地流转“小块变大块”，不断提升农业集约化、专业化、组织化和社会化水平。

加快推进城乡一体化进程。围绕建设“一小时经济圈”、“两小时经济带”，加快城乡交通路网规划建设。推动柳江、鹿寨与市区同城化发展，推进柳江县撤县建区，把穿山工业片区打造成为食品工业专业园区。加快融安打造市域次中心城市步伐。高标准修编沙塘镇总体规划，推进沙塘生态新区建设，打造新型城镇化先行先试区。深化城乡户籍制度改革，稳妥放开建制镇和县城落户限制，有序放开市区落户限制，稳步提高户籍人口城镇化水平。

加强农村基础设施建设。加大农村公路建设，推进融水、融安、三江石漠化区县乡公路改造，引导社会资本投资农村路网建设，实现行政村通水泥路率达80%。抓好水利基础设施建设，开工建设落久水利枢纽工程，积极推进洋溪水利枢纽工程前期工作，加快古偿河水库建设，实施各县城堤防及柳江支流整治工程，开展中小河流治理，除险加固水库55座，建设农田渠道420公里，恢复改善灌溉面积11万亩，解决15万农村人口饮水安全问题。推进融安、融水、三江风力发电项目建设，完成年度农村电网升级改造任务。抓好城乡风貌 改造、村寨防火改造和特色名镇名村建设，深入开展“美丽柳州·清洁乡村”活动，进一步改善农村人居环境。推进新一轮扶贫开发攻坚，抓好贫困地区基础设施建设，做好水库移民扶贫解困工作，增强贫困地区群众自我发展能力。

发展壮大县域经济。依托各县资源和产业现状，合理布局建设县域工业集中区，形成各具特色的县域产业发展格局。大力发展生态农业和中草药、竹木、茶叶、桑蚕等特色产业，加快发展民族风情旅游和观光休闲旅游，积极发展汽车、机械制造配套产业。

（四）加快构建现代服务体系，推动传统服务业向现代服务业转型立足优化布局、提升层次、完善功能，加快构建与工业升级、城市发展相适应的现代服务体系，推动产业结构优化升级。

加快推进重点项目建设。积极构建布局合理、功能齐备、协调发展的现代商贸体系，重点推进万达、华润、恒大、红星美凯龙等大型商贸项目建设。

完善现代物流体系，加快推进新柳邕农贸市场（一期）、柳东物流中心等项目，建设柳州汽车城生产性服务业发展示范区，提升大型物流园区集聚辐射能力，支持大型制造企业剥离和外包物流业务，发展壮大物流龙头企业。

促进城乡居民消费。借高速铁路建成之机，大力发展旅游购物、特色餐饮等产业，吸引外来消费。促进社区商业、信息咨询、家政养老、健康保健等便民服务，拉动居民消费。建设一批乡镇商贸中心和配送中心，开展社区生鲜农产品加工配送，改造一批乡镇集贸市场，扩大农村消费。加强市场价格监管，保持物价总水平基本稳定。全年服务业增加值力争增长10%以上。

打造旅游发展新优势。抓好列入自治区级重大旅游项目建设，启动百里柳江、元宝山旅游详规编制，加快建设百里柳江、三江程阳八寨5A级景区，积极推进融水至元宝山、三江至程阳八寨二级公路建设，把融水、三江打造成为广西旅游特色名县。支持大龙潭景区创建国家生态旅游示范区，建设柳北沙塘近郊生态休闲区。开发现代工业文明旅游，推进观光休闲旅游自驾车营地建设，完善旅游咨询、旅游标识等服务功能。加强旅游区域合作与国际交流，经桂林、广东、港澳台和东南亚等地“引客入柳”，不断提升柳州旅游的知名度。

大力发展新兴服务业。依托柳州国际会展中心，举办各类大型品牌展会，推动发展会展经济。大力发展电子商务，培育电子商务平台，引进大型电子商务企业来柳设立物流配送中心。实施“宽带柳州”战略，发展物联网服务、软件和服务外包等信息服务，提升信息服务业发展水平。积极促进“引金入柳”，吸引各类金融机构来柳设点；鼓励发展融资性担保公司、小额贷款公司、村镇银行；支持开展政、银、保联动的小微企业贷款保证保险，拓展中小微企业金融服务新领域；加强区域集优直接债务融资，加快推进柳州银行增资扩股；加大企业上市培育力度，推动柳药股份IPO上市和天涌科技、爱格富“新三板”挂牌，促进20亿元小微企业扶持债券发行。

（五）提升城市建设与管理水平，加快建设宜居宜业美丽柳州进一步加强城市建设与管理，启动较大的市申报工作，努力建设更加美丽柳州。

科学规划城市建设。开展空间优化、功能提升、生态保护三大行动，抓好城市总体规划实施评估，补充完善市区控制性详细规划，优化基础设施、产业发展、生态保护、公共服务、市民居住等空间布局；推进土地利用总体规划修编中期评估，优化国土空间开发。进一步完善城市功能分区，推进中心城区产业高端化和城市精品化，统筹规划现代商贸、科技服务、金融服务等产业；加强快环路沿线土地统筹开发，拓展和优化城市空间布局。

全面拓展对外交通。围绕建设区域性综合交通枢纽，打造便利交通，吸引更多人流、物流、资金流、信息流、技术流汇聚柳州。推动柳肇铁路加快开工建设，促成柳韶铁路规划落地，竣工贵广铁路（柳州段）。推进火车站站房改造、柳州东站还建等项目开工建设，加快推进柳州至梧州等5条续建高速公路建设，加快桂林至柳州至南宁高速公路改扩建；建设西江黄金水道项目、柳州港官塘作业区、鹧鸪江作业区泊位，扩建柳州白莲机场。

加快建设柳东新区。坚持产城融合发展，不断完善新区规划体系，制定实施景观风貌、建筑风格色彩等专项规划。实施230项新区项目建设，不断完善市政基础设施，竣工新柳大道延长线，加快贯通东外环柳东段、官塘大道南段等主骨架道路，进一步完善江滨区、核心区等片区路网，启动坪龙综合工程，推进柳东水厂、燃气管网等项目建设。配套完善公共事业设施，加快柳州国际会展中心（二期）、华侨城文化旅游科技体验园、文化广场、广西科技 大学汽车学院、职教园共享中心等项目建设，竣工鹿山学院（二期）、实验中学和实验小学等项目。

提高城市综合承载能力。编制完成《柳州市综合交通规划》，投资176亿元，推进160项市政重点工程建设。完善城市主骨架路网，推进环江滨水道路建设，竣工鹅山立交桥、西南出口道路跨铁路立交桥。推进城市中心区旧路改造及路网建设，实施交通“毛细血管”道路改造，改善市区交通环境。完善城市快速公交系统（BRT）线路，增设200个公共自行车租赁站点，开通水上公交巴士，做好水陆站点联通。大力推进阳和新村、火车南站周边、荣军路等片区旧城改造，启动旧城区排水防涝、竹鹅溪整治工程建设。加强供电、供气、供排水及通信等公共基础设施建设，推进500KV金沙江中游电站直流输电柳州工程、中缅天然气市区配套及管线工程建设。

抓好生态文明建设。绿化美化彩化城市，形成街道“一街一景”、公园“一园一品”、沿江“百里画廊”、石山“竞翠叠彩”的美丽景观。保护修复历史城区、历史街区，丰富历史文化名城内涵。积极创建国家环保模范城市，搞好节能减排，加强PM2.5监测，治理工业废气与烟尘、机动车尾气、施工扬尘，进一步提升空气环境质量。推进城郊污水收集工程、沙塘污水处理厂等设施建设，保护水体生态。加大天然气利用，推进公交车、出租车“油改气”，促进空气净化。

提高城市管理科学化水平。推进国家智慧城市试点建设，投入12.6亿元打造“智慧柳州”。完善“数字城管”平台，将绿化、环卫、城市照明等纳入监管范围。建立城市建设与管理长效机制，深入推进严厉打击违法占地违法建设“风暴行动”。清理整治违规户外广告和门店招牌，改善市容市貌。推进公交智能化，构建便捷、安全、智能、绿色的城市新型公共交通服务体系，创建国家“公交都市”示范城市。实施“畅通工程”，疏缓交通拥堵，让市民出行更加便捷、生活更加舒适。

（六）坚持保障和改善民生，让发展成果更好更公平惠及人民群众顺应人民群众对美好生活的新期待，切实保障和增进民生福祉，让发展成果最终体现到提高人民生活水平上来，体现到促进人的全面发展上来。

保持城乡居民收入持续增长。以提高城乡居民收入为工作着力点，以实现城乡居民收入倍增计划为目标，加大工资集体协商工作力度，进一步拓宽就业增收路径，多渠道增加财产性收入。大力推进全民创业，健全完善政策扶持、就业培训和创业服务机制，开发更多的就业岗位，强化就业和劳动技能培训。

引导劳动者转变就业观念，以促进创业带动就业，实施高校毕业生就业援助“4+1”计划，实现更高质量和更加充分的就业。全年城镇新增就业5.3万人，农村劳动力转移5万人。

不断增强社会保障能力。抓好社保扩面征缴，围绕被征地农民参加社会保险、社会保障卡发行等做好工作，提升社保覆盖范围和水平。完善新型农村合作医疗机制体制，提高新农合筹资和保障水平。建立和完善农村医疗救助经费即时结算管理平台，进一步规范城乡低保救助、医疗救助、农村五保供养等社会救助，提升救助能力和水平。加大对残疾人扶贫、就业服务和就业培训力度，积极开展残疾人帮扶救助。

抓好民生工程建设。今年为民办10件实事，主要是解决教育、扶贫、农产品质量安全等热点、难点问题，要把好事办好，实事办实。要加快安居工程建设，保障性住房新开工11558套，新增廉租住房补贴1000户，基本建成16000套，分配入住5000户。加大城市棚户区改造建设力度，扩大改制企业职工危旧房集中区改造范围，实施国有大企业危旧房改造工程，积极推动改造直管公房危旧房。推进城中村改造，开工建设柳北白沙村、柳南帽合村，加快改造鱼峰水南村、城中河东村、柳东新区下窑村。深入推进改造农村危房，全面完成三江县第二期村寨防火改造，继续实施全市农村住房统一保险及少数民族村寨火灾统保工程。抓好老有所养工程建设，加大养老设施建设力度，鼓励发展民办养老机构，增强社会养老服务能力和水平。

加强社会治理能力建设。深入创建“平安柳州”，实施“天网”工程五期，完善立体化、现代化社会治安防控体系；深化严打整治，加强社区警务建设和派出所基础建设。创新矛盾纠纷调处机制，将矛盾纠纷化解在萌芽状态。完善信访工作长效机制，探索网上受理信访制度，扎实开展领导干部“大接访”活动，切实解决群众实际问题。加强食品药品安全监管，建立覆盖全过程的食品药品监管制度，保障食品药品安全。

建立隐患排查治理和安全预防控制体系，遏制重特大安全事故。完善防灾减灾救灾、公共突发事件应急体系，提高公共突发事件应对能力。

（七）促进社会事业全面发展，不 断增强人民群众生活幸福感坚持统筹兼顾，把社会事业发展摆到更加突出位置，使之与经济发展同步推进，不断满足人民群众的新需求。

坚持优先发展教育。深入实施义务教育均衡发展学校基本建设三年攻坚计划，年内投资11.38亿元，推进19项重点教育工程建设；进一步优化学校布局调整，推进学校标准化建设，改造农村薄弱学校和中小学危旧房，不断改善义务教育学校办学条件，实现义务教育巩固率达到87%以上。提升学前教育普及率，规划建设一批公办乡镇中心幼儿园，实现学前教育三年毛入学率达到78%。加强示范高中和特色高中建设，扩大优质普通高中教育资源。强化职业院校基础能力建设，支持市二职校、交通学校创建国家中等职业教育改革发展示范学校，加快构建中高职有效衔接、城乡协调发展的职业教育体系。推进高等职业教育品牌建设，继续支持广西科技大学加快发展。健全家庭困难学生资助体系，支持特殊教育、民办教育加快发展。加强教师队伍建设，培养培训优秀教师，鼓励支持城镇学校教师支援农村和基础薄弱学校。

促进文化大发展大繁荣。坚持实施产业项目带动战略，推动文化产业大厦、中航航空文化体验园、柳州国际奇石文化产业园等项目建设，新建市群众艺术馆、柳宗元纪念馆，启动第三批民办博物馆建设。着力打造本土文化品牌，举办2014年中国柳州国际水上狂欢节。加快构建现代公共文化服务体系，新建一批村级公共服务中心、社区文化服务中心，支持“鱼峰歌圩”创建全国公共文化服务体系示范项目。推进文化惠民，做好“中外优秀剧目月月演”、“刘三姐大舞台周周演”、“水上大舞台天天演”，把广播电视“村村通”、“户户通”和社会电影公益放映惠及更多群众。加快东门城楼、曙光西路、鱼峰路南段周边历史文化街区保护与改造，支持三江侗族村寨申报世界文化遗产，做好侗族木构建筑及构造技艺的保护和传承。

统筹发展各项社会事业。加大人才资源开发力度，进一步完善人才政策体系。推进高层次创新创业人才开发，完成第三批市级人才小高地建设，建立6-8家人才小高地。巩固国家卫生城市创建成果，建立卫生城市管理长效机制。加快推进城乡卫生统筹，深入实施基本公共卫生服务项目，推广社区卫生服务机构家庭医生签约服务，全面推进公共卫生服务均等化。推行全国居民健康卡示范工程。完善卫生基础设施建设，投入资金6.15亿元推进市中医院迁建、第四人民医院综合楼等项目建设。做好疾病防控、妇幼卫生保健工作，加大艾滋病重点人群防治。推动全民健身运动广泛深入开展，大力发展群众体育和竞技体育。坚持计划生育基本国策，进一步完善计划生育利益导向机制，提高出生人口素质。

加强精神文明建设。深入贯彻党的十八大和十八届三中全会精神，弘扬中国特色社会主义和“中国梦”时代主旋律，全面提升精神文明建设水平。积极创建全国文明城市，扎实开展“双拥”共建和民兵预备役建设。推进民族团结进步模范区建设，巩固和发展平等团结互助和谐的社会主义民族关系。深入开展公民道德教育，弘扬真善美，传播正能量，构建和谐社会。

（八）加快政府职能转变，建设为民、务实、清廉的法治政府和服务型政府围绕开展作风建设年活动，加强政府自身建设，不断提高政府工作的科学化水平，不断提高政府的执行力和公信力。

全面正确履行政府职能。深化行政审批制度改革。进一步简政放权，减少行政审批环节，清理审批事项、收费项目、收费标准，提高依法管理经济和服务企业的能力。切实改进文风会风，精简会议和文件，深入基层和群众，加强调查和研究。认真开展党的群众路线教育实践活动，坚持市长“企业接待日”、“市民接待日”制度，帮助企业、群众解决实际困难和问题，进一步密切党群政群关系。加强政府执行力建设，健全行政责任体系，把各项工作落实到单位、部门和具体责任人，推动政府工作高效有序运转。严格绩效管理，优化绩效考评和绩效管理体系，促进行政效能不断提高。

加强法治政府建设。把严格依法办事贯穿于政府工作的各个环节，加强行政执法规范化建设，不断提高依法行政水平。依法扩大政务公开，做好政府信息发布工作，增加政府工作的透明度和公信力。强化政府内部层级监督，自觉接受人大依法监督、政协民主监督，关注和重视社会公众监督和新闻舆论监督，加强和改进行政监察与审计监督，依法依规履行各项职能。

牢固树立清正廉洁的政府形象。坚决贯彻执行《中央政治局关于改进工作作风、密切联系群众的八项规定》、《党政机关厉行节约反对浪费条例》、《党政机关国内公务接待管理规定》，按 照《建立健全惩治和预防腐败体系2013-2017年工作规划》要求，厉行勤俭节约，反对铺张浪费，开展“三公经费”预算公开，把有限的资源和财力用在推动发展和改善民生上。坚持标本兼治、综合治理、惩防并举、注重预防，认真落实廉政建设责任制，严肃查办违纪违法案件，纠正损害群众利益的不正之风，切实做到干部清正、政府清廉、政治清明。

各位代表！马蹄声疾催人奋进，东风劲吹鼓帆前行。党的十八届三中全会吹响了全面深化改革的号角，我们置身于这个伟大的改革时代，深感责任重大、使命光荣、信心百倍、前景光明，让我们紧密团结在以习近平同志为总书记的党中央周围，在市委的正确领导下，与全市人民一道，万众一心，奋发有为，乘势而上，为加快建设“五美五好”柳州、为努力完成历史赋予我们的崇高使命而奋勇前进！
